# In Vitro Activity of Ceftolozane-Tazobactam against *Escherichia coli*, *Klebsiella pneumoniae* and *Pseudomonas aeruginosa* Obtained from Blood Cultures from Sentinel Public Hospitals in South Africa

**DOI:** 10.3390/antibiotics12030453

**Published:** 2023-02-24

**Authors:** Olga Perovic, Ashika Singh-Moodley, Michelle Lowe

**Affiliations:** 1 Centre for Healthcare-Associated Infections, Antimicrobial Resistance and Mycoses, National Institute for Communicable Diseases a Division of the National Health Laboratory Service, Johannesburg 2192, South Africa; 2 Department of Clinical Microbiology and Infectious Diseases, School of Pathology, Faculty of Health Sciences, University of Witwatersrand, Johannesburg 2193, South Africa

**Keywords:** ceftolozane-tazobactam, *Escherichia coli*, *Klebsiella pneumoniae*, *Pseudomonas aeruginosa*, WGS

## Abstract

Multidrug-resistant (MDR) Gram-negative bacteria are responsible for the majority of healthcare-associated infections and pose a serious threat as they complicate and prolong clinical care. A novel cephalosporin-β-lactamase-inhibitor combination, ceftolozane-tazobactam (C/T) was introduced in 2014, which improved the treatment of MDR pathogens. This study aimed to evaluate the activity of C/T against *Escherichia coli* (n = 100), *Klebsiella pneumoniae* (n = 100), and *Pseudomonas aeruginosa* (n = 100) blood culture isolates in South Africa (SA). Isolates were sequentially selected (2010 to 2020) from the Group for Enteric, Respiratory, and Meningeal Diseases Surveillance (GERMS) programme in SA. Organism identification was performed using the matrix-assisted laser desorption/ionisation-time of flight mass spectrometry (MALDI-TOF MS) instrument (Microflex, Bruker Daltonics, Bremen, Germany), and antibiotic susceptibility was performed using the Sensititre instrument (Trek Diagnostic Systems, East Grinstead, UK). C/T resistance was reported in 16 *E. coli*, 28 *K. pneumoniae* and 13 *P. aeruginosa* isolates. Fifty percent of the C/T resistant isolates were subjected to whole-genome sequencing (WGS). According to the whole genome multilocus sequence typing (MLST) analysis, the *E. coli* isolates (n = 8) belonged to sequence type (ST)10, ST131, ST405, and ST410, the *K. pneumoniae* isolates (n = 14) belonged to ST1, ST37, ST73, ST101, ST231, ST307, ST336 and ST6065 (novel ST), and the *P. aeruginosa* isolates (n = 7) belonged to ST111, ST233, ST273, and ST815. The WGS data also showed that all the *E. coli* isolates harboured aminoglycoside (*aph* (3′′)-Ib, *aph* (6)-Id), macrolide (*mdf*A, *mph*A), and sulphonamide (*sul*2) antibiotic resistance genes, all the *K. pneumoniae* isolates harboured β-lactam (*bla*_CTX-M-15_), and sulphonamide (*sul*2) antibiotic resistance genes, and all the *P. aeruginosa* isolates harboured aminoglycoside (*aph* (3′)-IIb), β-lactam (*PAO*), fosfomycin (*fos*A), phenicol (*cat*B7), quinolone (*crp*P), and disinfectant (*qac*E) antibiotic resistance genes. It is evident that *E. coli*, *K. pneumoniae* and *P. aeruginosa* can adapt pre-existing resistance mechanisms to resist newer β-lactam molecules and inhibitors, since these isolates were not exposed to ceftolozane-tazobactam previously.

## 1. Introduction

*Escherichia coli*, *Klebsiella pneumoniae* and *Pseudomonas aeruginosa* are frequently isolated pathogens in the community and healthcare settings, and are the causes of infection-related morbidity and mortality [1,2]. Of concern is that these pathogens are becoming more resistant to available antibiotics [1].

A new cephalosporin-β-lactamase-inhibitor combination, ceftolozane/tazobactam (C/T) (sold under the brand name Zerbaxa, MERCK Connect, United States of America (USA)) was introduced in 2014 and has improved the treatment of carbapenem-resistant Gram-negative pathogens, especially *P. aeruginosa* [3,4]. Ceftazidime-avibactam (C/A), introduced in 2015 is also a combination antibiotic like C/T and consists of a broad-spectrum cephalosporin (i.e., ceftazidime) and a β-lactamase inhibitor (i.e., avibactam) [5].

C/T is a combination antibiotic that consists of a fifth-generation cephalosporin (ceftolozane) and a β-lactamase inhibitor (tazobactam) that exhibits bactericidal properties [6,7]. Ceftolozane, like other β-lactams, inhibits penicillin binding proteins (PBPs), which prevents bacterial cell wall synthesis and leads to cell death [1. Tazobactam inhibits most class A β-lactamases and some AmpC cephalosporinases (plasmid-mediated class C β-lactamases) and protects ceftolozane from hydrolysis [1,7]. C/T and C/A have been registered drugs in South Africa (SA) since 2022 [8]. Previously, these antibiotics were prescribed in SA as Section 21 drugs (i.e., before these antibiotics can be administered to patients, approval is needed from the hospital’s drug and therapeutic committee/s and the South African Health Products Regulatory Authority (SAHPRA)) [8,9]. These combination antibiotics are costly and not easily accessible in SA [9].

The use of C/T and C/A in SA is predominantly driven by the private healthcare sector [8]. The public healthcare sector in SA does not routinely offer susceptibility testing for C/T and C/A [9]. A comprehensive South African guide for new and existing antibiotics, which includes dose and administration strategies for critically ill patients has recently been published [8]. The focus on appropriate antibiotic stewardship practices is vital to maximise the efficacy and longevity of all new antibiotics that enter the healthcare sector [8].

Monitoring the susceptibility profile of these new combination antibiotics is important as increasing levels of resistance to C/T are observed, putting long-term use at risk [8].

This study aimed to evaluate the in vitro activities of C/T against *E. coli*, *K. pneumoniae* and *P. aeruginosa* and to molecularly characterise selected C/T resistant isolates using whole genome sequencing (WGS).

## 2. Results

### 2.1. Demographic Characteristics of Patients

*E. coli* (n = 100), *K. pneumoniae* (n = 100), and *P. aeruginosa* (n = 100) blood culture isolates were collected from 2010 to 2020. The median age for patients with *E. coli*, *K. pneumoniae* and *P. aeruginosa* bacteraemia was 36 years, 31 years and 44 years, respectively. The demographic characteristics of the patients are described in Table 1.

### 2.2. Phenotypic Characterisation of All Blood Culture Isolates

The antibiotic susceptibility results are shown in Table 2.

The six most active antibiotics against *E. coli* were C/A (susceptibility rate 96%), meropenem (susceptibility rate 91%), imipenem (susceptibility rate 89%), amikacin (susceptibility rate 89%), gentamicin (susceptibility rate 88%) and C/T (susceptibility rate 82%). Colistin showed intermediate resistance of 94%.

The six most active antibiotics against *K. pneumoniae* were CA (susceptibility rate 100%), meropenem (susceptibility rate 100%), imipenem (susceptibility rate 99%), amikacin (susceptibility rate 99%) and C/T (susceptibility rate 66%). Colistin showed intermediate resistance of 94%.

The six most active antibiotics against *P. aeruginosa* were amikacin (susceptibility rate 86%), C/T (susceptibility rate 85%), C/A (susceptibility rate 84%), gentamicin (susceptibility rate 78%), tobramycin (susceptibility rate 78%), and aztreonam (susceptibility rate 76%). Colistin showed intermediate resistance of 68%.

Thirty percent (30/100) of the *E. coli*, 61% (61/100) of the *K. pneumoniae*, and 24% (24/100) of the *P. aeruginosa* isolates were multidrug resistant (MDR).

### 2.3. WGS of Selected Blood Culture Isolates

Half (50%) of the C/T resistant *E. coli* (n = 8/17), *K. pneumoniae* (n = 14/28), and *P. aeruginosa* (n = 7/13) isolates were subjected to WGS (Table A1).

#### 2.3.1. *E. coli* Isolates

The average genome size of the sequenced *E. coli* isolates was 5035 kb with a coverage depth of 68 × to 133 ×. The average G + C content was 50.

The *E. coli* isolates belonged to four different sequence types (STs) (Table 3). The *E. coli* isolates harboured between one to seven β-lactamase genes. In total, 138 antibiotic resistance genes were found in the sequenced *E. coli* isolates. All the *E. coli* isolates harboured the *aph* (3′′)-Ib, *aph* (6)-Id, *mdf*A, *mph*A and *sul*2 genes (Table 3). The majority of the isolates harboured the *aac* (6′)-Ib-cr, *aad*A5, *bla*_CTX-M-15_, *bla*_OXA-1_, *drf*A17 and *sul*1. No *Amp*C-genes (also known as *Pseudomonas*-derived cephalosporinase (PDC) genes) were detected. All the *E. coli* isolates harboured the *Inc*FIB (AP001918) and *Inc*FIA plasmids (Table 4).

#### 2.3.2. *K. pneumoniae* Isolates

The average genome size of the sequenced *K. pneumoniae* isolates was 5611 kb with a genome coverage of 32× to 125×. The average G + C content was 57.

The *K. pneumoniae* isolates belonged to eight different STs of which ST6065 is a novel ST (i.e., a newly assigned ST by the BIGSdb-Pasteur database) (Table 3). The *K. pneumoniae* isolates harboured between one to 14 β-lactamase genes. In total, 312 antibiotic resistance genes were found in the sequenced *K. pneumoniae* isolates. All *K. pneumoniae* isolates harboured the *bla*_CTX-M-15_, and *sul*2 genes (Table 3). The majority of the isolates harboured the *aph* (3′′)-Ib, *aph* (6)-Id, *bla*OXA-1, *bla*TEM-1B, *fos*A, *cat*B3, *Oqx*A and *Oqx*B genes. No *Amp*C-genes were detected. The majority of the *K. pneumoniae* isolates harboured the *Inc*FII (K) (n = 13), *Inc*Fib (K) (n = 12) and *Inc*R (n = 11) plasmids (Table 4).

#### 2.3.3. *P. aeruginosa* Isolates

The average genome size of the sequenced *P. aeruginosa* isolates was 6974 kb with a genome coverage of 57× to 84×. The average G + C content was 50.

The *P. aeruginosa* isolates belonged to four different STs (Table 3). The *P. aeruginosa* isolates harboured between one to seven β-lactamase genes. In total, 99 antibiotic resistance genes were found in the sequenced *P. aeruginosa* isolates. All *P. aeruginosa* isolates harboured the *aph* (3′)-Iib, PAO, *fos*A, *cat*B7, *crp*P and *qac*E genes (Table 3). The majority of the isolates harboured the *aad*A2, *aac* (6′)-II, *bla*_VIM-2_, *dfr*B5 and *sul*2 genes. No *Amp*C-genes were detected. No plasmids were detected in the sequenced *P. aeruginosa* isolates (Table 4).

All the sequenced *E. coli*, *K. pneumoniae* and *P. aeruginosa* isolates were resistant to cephalosporins (cefotaxime and ceftazidime) (Table A2). All the *E. coli* and *K. pneumoniae* isolates were resistant to aztreonam except for most of the *P. aeruginosa* isolates, which were shown to be susceptible. All *E. coli* and *P. aeruginosa* isolates were resistant to one or more tested carbapenems. However, all the sequenced *K. pneumoniae* isolates were susceptible to all tested carbapenems.

## 3. Discussion

With the continuous rise of multidrug resistance in *K. pneumoniae* and *P. aeruginosa*, and to a lesser extent in *E. coli* there are only a few treatment options left for infected patients [7]. With the introduction of C/T in 2014, it has proven to be highly effective against MDR Gram-negative pathogens. The in vitro activity of C/T, against *E. coli*, *K. pneumoniae*, and *P. aeruginosa* isolates obtained from blood cultures from sentinel public hospitals in SA were investigated. The majority of the isolates were collected from adult patients in the Gauteng province; this province has the highest population in South Africa [10].

This study showed that the majority of the *E. coli*, and *P. aeruginosa* isolates are highly susceptible to C/T (82% to 85%). In comparison, *K. pneumoniae* isolates indicated decreased susceptibility towards C/T (66%). Findings from a previous study showed similar C/T susceptibility rates to *E. coli*, *K. pneumoniae*, and *P. aeruginosa* [7]. In contrast, higher C/T susceptibility rates in *K. pneumoniae* were reported in Germany and the USA [1,11]. The decreased susceptibility to C/T in the *K. pneumoniae* isolates reported in this study could potentially be due to increased antibiotic resistance [7]. In this study, we have detected multiple β-lactamase genes in the sequenced *K. pneumoniae* isolates (1 to 14 β-lactamase genes per isolate).

Interesting to note is that all the isolates were highly susceptible to C/A (84% to 100%). The tested *E. coli* isolates showed ≥80% susceptibility towards amikacin, imipenem and meropenem, *K. pneumoniae* isolates showed ≥80% susceptibility towards amikacin, ertapenem, imipenem and meropenem, and *P. aeruginosa* isolates showed ≥80% susceptibility towards amikacin. All *E. coli* (94%), *K. pneumoniae* (94%), and *P. aeruginosa* (68%) isolates showed intermediate resistance to colistin.

In this study, multidrug resistance was detected in 30% of the *E. coli* isolates, 61% of the *K. pneumoniae* isolates, and 24% of the *P. aeruginosa* isolates (Table 2). C/T was not effective against the majority of MDR isolates detected in this study. In contrast, other studies reported that treatment with C/T generally led to favorable clinical outcomes among patients with MDR, extensively drug-resistant (XDR) or pan drug-resistant (PDR) bloodstream infections associated with *E. coli*, *K. pneumoniae*, and *P. aeruginosa* [12,13,14,15].

WGS is a powerful tool that can be used to characterise the genetic diversity of bacterial populations and can also be used for the prediction of bacterial antibiotic resistance profiles [16]. However, the prediction of susceptibility or resistance to antibiotics based only on the presence or absence of previously known genes is still under investigation and discordances are reported in the literature [16]. Half of the C/T resistant isolates were subjected to sequencing for genomic characterisation. *Amp*C (*PDC*) and *bla*_GES_ hyper-production are factors linked to C/T resistance [3,4,17,18,19,20]. However, the *bla*_GES_ and *Amp*C genes were not detected in our isolate collection that was subjected to WGS. Other antibiotic resistant genes reported in C/T resistant isolates are the *bla*_CTX-M_, and *bla*_SHV_ genes [18]. The *bla*_CTX-M-15_ gene was detected in the majority of the *E. coli* isolates, and in all the *K. pneumoniae* isolates. The *bla*_SHV_ genes were only detected in *K. pneumoniae* isolates. The precise resistance mechanisms leading to C/T resistance could not be determined. Further studies are required to assess the expression and functionality of the detected genes in the studied isolate population to predict the phenotype consequences of the C/T resistant genotype.

Limitations of the study: (i) C/T was only evaluated for blood cultures, (ii) isolates and data originated from sentinel public hospitals in SA, (iii) small sample size, and (iv) not all the C/T resistant isolates could be sequenced due to limited funding.

## 4. Materials and Methods

### 4.1. Study Setting

A total of 100 *E. coli*, 100 *K. pneumoniae*, and 100 *P. aeruginosa* blood culture isolates were chronologically selected from storage (−70 °C). A three-week exclusion period was applied to avoid duplicate isolates of the same organism from the same patient.

The isolates were initially collected for the Group for Enteric, Respiratory, and Meningeal Diseases Surveillance (GERMS) program in SA (2010 to 2020), which receives clinical isolates from sentinel sites in the Free State, Gauteng, KwaZulu-Natal, Eastern- and Western Cape provinces. Demographic and clinical information of patients was collected by surveillance officers through medical record reviews and/or patient interviews using standard case report forms (CRFs). All GERMS-SA isolates were initially processed and stored as followed: the bacterial cultures were grown on blood or chocolate agar plates (Diagnostic Media Products (DMP), National Health Laboratory Service (NHLS), Johannesburg, SA) for no more than 18–27 h. The agar plates were carefully inspected for any contaminating bacterial or fungal colonies. Using a sterile disposable Pasteur pipette, 1 mL of TSB + 10% glycerol (DMP, NHLS, Johannesburg, SA) was dispensed into cryovials. Using a sterile swab or loop, a heavy sweep of growth was taken and emulsified in the cryovial containing the TSB + 10% glycerol. The cryovials were tightly sealed and placed in allocated cryoboxes and immediately stored at −70 °C.

### 4.2. Phenotypic Characterisation

All organisms were processed by the Centre for Healthcare-associated infections, Antimicrobial Resistance and Mycoses (CHARM), National Institute for Communicable Diseases (NICD), a division of the NHLS, Johannesburg, SA. The selected GERMS-SA isolates were retrieved from −70 °C storage. A loop full of the isolate + TSB + 10% glycerol mixture was streaked and grown on blood agar plates (DMP, NHLS, Johannesburg, SA) for no more than 18 h to 24 h. The agar plates were carefully inspected for any contaminating bacterial or fungal colonies. Organism identification was confirmed using matrix-assisted laser desorption/ionisation-time of flight mass spectrometry (MALDI-TOF MS) (Microflex, Bruker Daltonics, Bremen, Germany). All MALDI-TOF MS score values were between 2.00 to 3.00 (high confidence identification) and fell in the consistency category A (high consistency). Antimicrobial susceptibility testing (AST) was performed using the Sensititre instrument (Trek Diagnostic Systems, East Grinstead, UK) with the commercially available Gram-negative DKMGN panel (Separation Scientific, Johannesburg, SA) which included amikacin, amoxicillin/clavulanic acid, aztreonam, cefotaxime, ceftazidime, C/A, C/T, ciprofloxacin, colistin, ertapenem, gentamicin, imipenem, meropenem, tigecycline, tobramycin, and trimethoprim/sulfamethoxazole. The AST results were interpreted using the 2021 Clinical Laboratory Standards Institute (CLSI) guidelines (no interpretation for tigecycline was provided) [21]. MDR isolates were defined as non-susceptibility to one or more antibiotic agents in three or more antimicrobial classes [22]. XDR isolates were defined as non-susceptibility to at least one agent in all but two or fewer antimicrobial categories (i.e., bacterial isolates remain susceptible to only one or two categories), and PDR isolates were defined as non-susceptible to all agents in all antimicrobial categories [22].

### 4.3. Molecular Characterisation

Half (50%) of the C/T resistant isolates were randomly selected for WGS (i.e., 8 *E. coli*, 14 *K. pneumoniae*, and 7 *P. aeruginosa* isolates). The genomic DNA (gDNA) of the selected isolates was extracted with the QIAamp mini kit (Qiagen, Hilden, Germany) with the inclusion of lysozyme (10 mg/mL; Sigma-Aldrich, MS, USA) to ensure sufficient lysis. The quantity of the extracted gDNA was determined on Qubit 4.0 (Thermo Scientific, Waltham, MA, USA). Multiplexed paired-end libraries were prepared using the Nextera DNA Prep kit, followed by sequencing (2 × 150 bp) on a NextSeq 550 instrument (Illumina, Inc., San Diego, CA, USA) with 100× coverage at the NICD Sequencing Core Facility, Johannesburg, SA. Raw paired-end reads were analysed using the Jekesa pipeline (v1.0; https://github.com/stanikae/jekesa (accessed on 27 January 2022)).

Briefly, Trim Galore! (v0.6.2; https://github.com/FelixKrueger/TrimGalore (accessed on 27 January 2022)) was used to filter the paired-end reads (Q > 30 and length > 50 bp) [23]. *De novo* assembly was performed using SKESA v2.3.0 (https://github.com/ncbi/SKESA/releases (accessed on 27 January 2022)) and the assembled contigs were polished using Shovill (v1.1.0; https://github.com/tseemann/shovill (accessed on 27 January 2022)) [24,25]. Assembly metrics were calculated using QUAST (v5.0.2; http://quast.sourceforge.net/quast (accessed on 27 January 2022)) [26]. The assembled genome files were submitted to the National Center for Biotechnology Information GenBank and are available under BioProject number: PRJNA819852.

The multilocus sequence typing (MLST) profiles were determined using the MLST tool (version 2.16.4; https://github.com/tseemann/mlst (accessed on 20 September 2022)) [27]. Antimicrobial resistance (AMR) gene search was performed using ABRicate (version 1.0.1; https://github.com/tseemann/ABRicate (accessed on 20 September 2022)), against the Comprehensive Antibiotic Resistance Database (CARD), CARD-prevalence, Virulence Factor Database (VFDB) and ResFinder—Center for Genomic Epidemiology (CGE) database, with a gene alignment coverage cut-off of ≥95% and blastn sequence similarity of ≥95% [28,29,30,31,32,33].

### 4.4. Statistical Analysis

Microsoft Excel (version 2016) was used for data entry and basic statistical analysis (medians, interquartile ranges and percentiles). Additional data analyses were carried out using STATA statistical software package (version 14; StataCorp LP, USA).

## 5. Conclusions

Antibiotic resistance is a global health threat that limits the optimal care of patients infected with healthcare-associated pathogens. The increasing antibiotic resistance should be closely monitored. Since these isolates were not exposed to C/T previously, it is evident that *E. coli*, *K. pneumoniae*, and *P. aeruginosa* can adapt pre-existing resistance mechanisms, such as the production of catalytic enzymes (i.e., ESBLs and carbapenemases), altered PBPs, reduction in outer membrane channels resulting in decreased porin activity influx/expression, and an increase in efflux pumps to resist β-lactam molecules and inhibitors. In this study, C/T was not effective against the majority of MDR isolates.

## Figures and Tables

**Table 1:** Demographic characteristics of patients with *Escherichia coli*, *Klebsiella pneumoniae* and *Pseudomonas aeruginosa* bacteraemia.

Demographic Characteristics	*E. coli*	*K. pneumoniae*	*P. aeruginosa*
	n = 100	n = 100	n = 100
Age			
Median age (interquartile range (IQR))	36 years (21–54 years)	31 years (130 days–51 years)	44 years (11–58 years)
Sex			
Female	61	36	41
Male	37	60	55
Unknown	2	4	4
Ward			
Adult	74	57	69
Paediatric	24	40	25
Unknown	2	3	6
Province			
Eastern Cape	1	-	-
Free State	-	10	2
Gauteng	98	65	46
Western Cape	-	24	41
KwaZulu-Natal	1	1	10
Unknown	-	-	1

**Table 2:** Antibiotic susceptibility profiles of 100 *Escherichia coli*, 100 *Klebsiella pneumoniae* and 100 *Pseudomonas aeruginosa* blood culture isolates.

		Amoxicillin/Clavulanate acid	Ceftazidime/Avibactam	Ceftolozane/Tazobactam	Piperacillin/Tazobactam	Cefotaxime	Ceftazidime	Aztreonam	Ertapenem	Imipenem	Meropenem	Colistin	Gentamicin	Tobramycin	Amikacin	Ciprofloxacin	Trimethoprim/Sulfamethoxazole	MDR Detected
*E. coli*	S	36	96	82	77	62	70	61	79	89	91	0	88	74	89	44	25	30
	I	18	0	2	1	0	5	4	2	3	1	94	0	2	7	8	0	
	R	46	4	16	22	38	25	35	19	8	8	6	12	24	4	48	75	
*K. pneumoniae*	S	33	100	66	51	35	36	36	97	99	100	0	41	39	99	43	34	61
	I	1	0	6	13	0	2	0	2	1	0	94	0	10	1	5	0	
	R	66	0	28	36	64	62	64	1	0	0	6	59	51	0	52	66	
*P. aeruginosa*	S	0 *	84	85	67	0 *	75	76	0 *	65	71	0	78	78	86	73	0 *	24
	I	0 *	0	2	10	0 *	6	11	0 *	8	9	68	3	2	2	1	0 *	
	R	0 *	16	13	23	0 *	19	13	0 *	27	20	32	19	20	12	26	0 *	

S = Susceptible; I = Intermediate; R = Resistant; MDR = Multidrug resistance; * = Antibiotic MIC breakpoints not covered in the 2021 Clinical Laboratory Standards Institute (CLSI) guidelines.

**Table 3:** Antibiotic resistance genes detected in the sequenced *Escherichia coli*, *Klebsiella pneumoniae* and *Pseudomonas aeruginosa* blood culture isolates.

Antibiotic Class		Organism	Antibiotic Resistance Gene	n =	ST
Aminoglycoside		*E. coli*	*aad*A5	7	131; 405; 410
			*aac* (3)-IIa	1	10
			*aac* (3)-Iid	1	405
			*aac* (6′)-Ib-cr	7	10; 405; 410
			*aph* (3′′)-Ib; *aph* (6)-Id	8	10; 131; 405; 410
			*rmt*B	1	405
		*K. pneumoniae*	*aac* (3)-Iia	13	37; 73; 101; 231; 307; 336; 6065 *
			*aac* (6′)-Ib-cr	10	73; 101; 231; 307; 336; 6065 *
			*aad*A1	6	37; 73; 101; 307; 336
			*aad*A16	5	231; 307
			*aad*A2	1	6065 *
			*aph* (3′′)-Ib	11	37; 73; 101; 231; 307; 336
			*aph* (6)-Id	12	37; 73; 101; 231; 307; 336
			*aac* (3)-Iid	2	1; 336
		*P. aeruginosa*	*aac* (3)-Id	2	233; 815
			*aac* (6′)-29a	2	111
			*aac* (6′)-Ib3; *aac* (6′)-Ig; *aac* (6′)-Iic	1	273
			*aac* (6′)-Il; *aad*A2	4	233; 815
			*aph* (3′’)-Ib	2	233; 273
			*aph* (3′)-Iib	7	111; 233; 273; 815
			*aph* (6)-Id	3	233; 273
β-lactam		*E. coli*	*bla*_CMY-2_	5	10; 410
			*bla*_CTX-M-15_	7	10; 405; 410
			*bla*_CTX-M-27_	1	131
			*bla*_NDM-5_	1	405
			*bla*_OXA-1_	7	10; 405; 410
			*bla*_OXA-181_	4	410
			*bla*_TEM-1B_	6	405; 410
		*K. pneumoniae*	*bla*_CTX-M-15_	14	37; 73; 101; 231; 307; 336; 6065 *
			*bla*_CMY-4_	1	307
			*bla*_OXA-1_	11	37; 73; 101; 307; 336; 6065 *
			*bla*_OXA-9_	4	37; 101; 307
			*bla*_SCO-1_	4	73; 307; 336
			*bla*_SHV-1_; *bla*_SHV-26_	1	73
			*bla*_SHV-28_	8	101; 231; 307
			*bla*_SHV-36_	1	6065 *
			*bla*_SHV-40_; *bla*_SHV-56_; *bla*_SHV-79_; *bla*_SHV-85_; *bla*_SHV-89_	1	37
			*bla*_SHV-94_; *bla*_SHV-96_; *bla*_SHV-172_	2	336
			*bla*_SHV-78_; *bla*_SHV-98;_*bla*_SHV-145_; *bla*_SHV-161_; *bla*_SHV-179_; *bla*_SHV-194_; *bla*_SHV-199_	1	73
			*bla*_SHV-106_	8	101; 231; 307
			*bla*_SHV-187_	1	1
			*bla*_TEM-1A_	1	101
			*bla*_TEM-1B_	12	1; 37; 73; 231; 307; 336; 6065 *
			*bla*_TEM-1C_	1	6065
		*P. aeruginosa*	*bla*_NPS_	1	273
			*bla*_OXA-4_	3	273
			*bla*_OXA395_	3	111; 815
			*bla*_OXA-485_; *bla*_OXA-488_	1	273
			*bla*_OXA-486_	3	273
			*bla*_PAO_	7	111; 233; 273; 815
			*bla*_VIM-2_	6	111; 233; 815
Fosfomycin		*E. coli*	None detected	-	-
		*K. pneumoniae*	*fos*A	13	1; 37; 73; 101; 231; 307; 336
		*P. aeruginosa*	*fos*A	7	111; 233; 273; 815
Macrolide		*E. coli*	*mdf*A; *mph*A	8	10; 131; 405; 410
		*K. pneumoniae*	*ere*A	2	73; 307
			*mph*A	2	336; 6065 *
		*P. aeruginosa*	None detected	-	-
Phenicol		*E. coli*	*cat*B3	7	10; 405; 410
		*K. pneumoniae*	*cat*A1	1	336
			*cat*A2	8	1; 101; 307; 336; 6065 *
			*cat*B3	11	37; 101; 307; 336; 6065 *
			*cml*A1	3	37; 73; 307
			*flo*R	1	307
		*P. aeruginosa*	*cat*B7	7	111; 233; 273; 815
			*cml*A1	3	233
Quinolone		*E. coli*	*aac* (6′)-Ib-cr	7	10; 405; 410
			*qnr*S1	6	10; 405; 410
		*K. pneumoniae*	*Oqx*A; *Oqx*B	13	1; 37; 73; 101; 231; 0307; 336
			*aac* (6′)-Ib-cr	11	73; 101; 231; 307; 336; 6065 *
			*qnr*B1; *qnr*S1	1	37
			*qnr*B6	4	231; 307
		*P. aeruginosa*	*aac* (6′)-Ib-cr	1	273
			*crp*P	7	111; 233; 273; 815
Rifampicin		*E. coli*	None detected	-	-
		*K. pneumoniae*	*ARR*-2	2	37; 73
			*ARR*-3	4	231; 307
		*P. aeruginosa*	*ARR*-5	2	233
Sulphonamide		*E. coli*	*sul*1	7	131; 405; 410
			*sul*2	8	10; 131; 405; 410
		*K. pneumoniae*	*sul*1	10	37; 73; 101; 231; 307; 336; 6065 *
			*sul*2	14	1; 37; 73; 101; 231; 307; 336; 6065 *
		*P. aeruginosa*	*sul*1	6	111; 233; 273; 815
Tetracycline		*E. coli*	*tet*A	2	131; 405
			*tet*B	5	405; 410
		*K. pneumoniae*	*tet*A	2	37; 336
			*tet*D	7	73; 101; 307
		*P. aeruginosa*	*tet*G	4	233; 273
Trimethoprim		*E. coli*	*dfr*A14	5	10; 410
			*dfr*A17	7	131; 405; 410
		*K. pneumoniae*	*dfr*A1	1	101
			*dfr*A12	1	6065 *
			*dfr*A14	8	37; 73; 101; 307; 336
			*dfr*A15	1	336
			*dfr*A27	5	231; 307
			*dfr*A30	2	1; 336
		*P. aeruginosa*	*dfr*B5	4	233; 815
Disinfectant		*E. coli*	*sit*ABCD	4	10; 131; 405
			*qac*E	7	131; 405; 410
		*K. pneumoniae*	*Oqx*A; *Oqx*B	13	1; 37; 73; 101; 231, 307; 336
			*qac*E	10	37; 73; 101; 231; 307; 336; 6065 *
		*P. aeruginosa*	*qac*E	7	111; 233; 273; 815

ST = Sequence type; * = Novel ST detected.

**Table 4:** Plasmids detected in the sequenced *Escherichia coli*, *Klebsiella pneumoniae* and *Pseudomonas aeruginosa* blood culture isolates.

Organism	ST	Plasmid	Total Number of Isolates
*E. coli*	10	*Inc*FIB (AP001918); ColRNAI; Col (MG828); *Inc*FIA; *Inc*X4; *Inc*FII; *Inc*I1	1
	131	*Inc*FII (pRSB107); *Inc*FIB (AP001918); ColRNAI; Col156; Col (MG828); *Inc*FIA;	1
	405	*Inc*FII (pAMA1167-NDM-5) *; *Inc*FIB (AP001918); Col (MG828) *; *Inc*FIA; p0111 *; *Inc*FII *; *Inc*Q1 *	2
	410	*Inc*FII (pRSB107); *Inc*B/O/K/Z; *Inc*FII (pAMA1167-NDM-5); *Inc*FIB (AP001918); *Inc*FIA; Col (BS512); ColKP3; *Inc*X4; IncX3; *Inc*FII (pCoo)	4
*K. pneumoniae*	1	*Inc*FII (K); *Inc*R; *Inc*FIA (HI1)	1
	37	*Inc*FII (K); Col440I; *Inc*FIB (K); ColRNAI; *Inc*FII	1
	73	*Inc*FII (K); Col440I; *Inc*R; *Inc*FIB (K); *Inc*FIA (HI1)	1
	101	*Inc*FII (K); *Inc*R; *Inc*FIB (K); Col440II; *Inc*FII (pCRY) *; ColRNAI *	2
	231	*Inc*FII (K); Col440I; *Inc*R; *Inc*FIB (K)	1
	307	*Inc*FII (K); Col440I *; *Inc*R *; *Inc*Fib (K); *Inc*FIB (pQil) *; *Inc*FIA (HI1) *; ColRNAI *; Col (MG828) *; *Inc*N *; *Inc*A-C2 *	5
	336	*Inc*FII (K); *Inc*R; *Inc*FIB (K); *Inc*FIB (pQil) *; ColRNAI *; *Inc*FIB (Mar) *; *Inc*FII *; *Inc*HI1B *; *Inc*Q1 *	2
	6065	Col440I; *Inc*Fib (Mar); *Inc*FII (pKPX1)	1
*P. aeruginosa*	ST111	-	0
	ST233	-	0
	ST273	-	0
	ST815	-	0

* = Plasmids not detected in all isolates; - = No plasmid/s detected.

## Data Availability

The datasets presented in this study can be found in the article or in the Appendix A.

## Appendix A

**Table A1:** Antibiotic resistance genes and plasmids detected in the sequenced *Escherichia coli*, *Klebsiella pneumoniae*, and *Pseudomonas aeruginosa* blood culture isolates.

Isolate ID		ESCCO-ML0045	ESCCO-016	ESCCO-10882	ESCCO-ML0129	ESCCO-10151	ESCCO-10377	ESCCO-9385	ESCCO-9889	KLEPN-0028	KLEPN-0203	KLEPN-0098	KLEPN-0024	KLEPN-0196	KLEPN-0004	KLEPN-0051	KLEPN-0062	KLEPN-0063	KLEPN-0109	KLEPN-0192	KLEPN-0036	KLEPN-0175	KLEPN-0097	PSEAE-7954	PSEAE-7985	PSEAE-7981	PSEAE-8174	PSEAE-8183	PSEAE-7986	PSEAE-7904
Year		2012	2020	2016	2013	2015	2015	2016	2016	2010	2010	2010	2010	2010	2010	2010	2010	2010	2010	2010	2010	2010	2010	2014	2014	2014	2014	2014	2014	2014
ST		10	131	405	405	410	410	410	410	1	37	73	101	101	231	307	307	307	307	307	336	336	6065	111	111	233	233	233	273	815
Province		GA	GA	GA	GA	GA	GA	GA	GA	WC	KZ	WC	GA	GA	WC	GA	GA	GA	GA	GA	WC	GA	GA	GA	WC	GA	GA	WC	GA	GA
Ward		A	A	A	A	A	A	A	A	P	P	A	A	A	A	P	A	P	A	P	P	P	P	A	A	A	P	A	P	U
Antibiotic resistance genes	*aad*A1	N	N	N	N	N	N	N	N	N	Y	Y	Y	Y	N	N	Y	N	N	N	N	Y	N	N	N	N	N	N	N	N
	*aad*A2	N	N	N	N	N	N	N	N	N	N	N	N	N	N	N	N	N	N	N	N	N	Y	N	N	Y	Y	Y	N	Y
	*aad*A5	N	Y	Y	Y	Y	Y	Y	Y	N	N	N	N	N	N	N	N	N	N	N	N	N	N	N	N	N	N	N	N	N
	*aad*A16	N	N	N	N	N	N	N	N	N	N	N	N	N	Y	Y	Y	N	Y	Y	N	N	N	N	N	N	N	N	N	N
	*aac* (3)-IIa	Y	N	N	N	N	N	N	N	N	Y	Y	Y	Y	Y	Y	Y	Y	Y	Y	Y	Y	Y	N	N	N	N	N	N	N
	*aac* (3)-Id	N	N	N	N	N	N	N	N	N	N	N	N	N	N	N	N	N	N	N	N	N	N	N	N	N	Y	N	N	Y
	*aac* (3)-IId	N	N	N	Y	N	N	N	N	Y	N	N	N	N	N	N	N	N	N	N	Y	N	N	N	N	N	N	N	N	N
	*aac* (6′)-Ib-cr	Y	N	Y	Y	Y	Y	Y	Y	N	N	Y	N	Y	Y	Y	N	Y	Y	Y	Y	Y	Y	N	N	N	N	N	N	N
	*aac* (6′)-29a	N	N	N	N	N	N	N	N	N	N	N	N	N	N	N	N	N	N	N	N	N	N	Y	Y	N	N	N	N	N
	*aac* (6′)-Ib3	N	N	N	N	N	N	N	N	N	N	N	N	N	N	N	N	N	N	N	N	N	N	N	N	N	N	N	Y	N
	*aac* (6′)-Ig	N	N	N	N	N	N	N	N	N	N	N	N	N	N	N	N	N	N	N	N	N	N	N	N	N	N	N	Y	N
	*aac* (6′)-Il	N	N	N	N	N	N	N	N	N	N	N	N	N	N	N	N	N	N	N	N	N	N	N	N	Y	Y	Y	N	Y
	*aac* (6′)-IIc	N	N	N	N	N	N	N	N	N	N	N	N	N	N	N	N	N	N	N	N	N	N	N	N	N	N	N	Y	N
	*aph* (3′′)-Ib	Y	Y	Y	Y	Y	Y	Y	Y	N	Y	Y	Y	Y	Y	Y	Y	Y	Y	Y	N	Y	N	N	N	N	N	N	N	N
	*aph* (3′)-IIb	N	N	N	N	N	N	N	N	N	N	N	N	N	N	N	N	N	N	N	N	N	N	Y	Y	Y	Y	Y	Y	Y
	*aph* (3′′)-Ib	N	N	N	N	N	N	N	N	N	N	N	N	N	N	N	N	N	N	N	N	N	N	N	N	N	N	Y	Y	N
	*aph* (6)-Id	Y	Y	Y	Y	Y	Y	Y	Y	N	Y	Y	Y	Y	Y	Y	Y	Y	Y	Y	Y	Y	N	N	N	Y	N	Y	Y	N
	*rmt*B	N	N	Y	N	N	N	N	N	N	N	N	N	N	N	N	N	N	N	N	N	N	N	N	N	N	N	N	N	N
	*bla*_CMY-2_	Y	N	N	N	Y	Y	Y	Y	N	N	N	N	N	N	N	N	N	N	N	N	N	N	N	N	N	N	N	N	N
	*bla*_CMY-4_	N	N	N	N	N	N	N	N	N	N	N	N	N	N	N	N	N	N	Y	N	N	N	N	N	N	N	N	N	N
	*bla*_CTX-M-15_	Y	N	Y	Y	Y	Y	Y	Y	Y	Y	Y	Y	Y	Y	Y	Y	Y	Y	Y	Y	Y	Y	N	N	N	N	N	N	N
	*bla*CTX-M-27	N	Y	N	N	N	N	N	N	N	N	N	N	N	N	N	N	N	N	N	N	N	N	N	N	N	N	N	N	N
	*bla*NDM-5	N	N	Y	N	N	N	N	N	N	N	N	N	N	N	N	N	N	N	N	N	N	N	N	N	N	N	N	N	N
	*bla*NPS	N	N	N	N	N	N	N	N	N	N	N	N	N	N	N	N	N	N	N	N	N	N	N	N	N	N	N	Y	N
	*bla*OXA-1	Y	N	Y	Y	Y	Y	Y	Y	N	Y	Y	Y	Y	N	Y	Y	Y	N	Y	Y	Y	Y	N	N	N	N	N	N	N
	*bla*OXA-4	N	N	N	N	N	N	N	N	N	N	N	N	N	N	N	N	N	N	N	N	N	N	N	N	Y	Y	Y	N	N
	*bla*OXA-9	N	N	N	N	N	N	N	N	N	Y	N	Y	Y	N	N	Y	N	N	N	N	N	N	N	N	N	N	N	N	N
	*bla*OXA-181	N	N	N	N	Y	Y	Y	Y	N	N	N	N	N	N	N	N	N	N	N	N	N	N	N	N	N	N	N	N	N
	*bla*OXA-395	N	N	N	N	N	N	N	N	N	N	N	N	N	N	N	N	N	N	N	N	N	N	Y	Y	N	N	N	N	Y
	*bla*OXA-485	N	N	N	N	N	N	N	N	N	N	N	N	N	N	N	N	N	N	N	N	N	N	N	N	N	N	N	Y	N
	*bla*OXA-486	N	N	N	N	N	N	N	N	N	N	N	N	N	N	N	N	N	N	N	N	N	N	N	N	Y	Y	Y	N	N
	*bla*OXA-488	N	N	N	N	N	N	N	N	N	N	N	N	N	N	N	N	N	N	N	N	N	N	N	N	N	N	N	Y	N
	*bla*PAO	N	N	N	N	N	N	N	N	N	N	N	N	N	N	N	N	N	N	N	N	N	N	Y	Y	Y	Y	Y	Y	Y
	*bla*SCO-1	N	N	N	N	N	N	N	N	N	N	Y	N	N	N	N	Y	N	Y	N	N	Y	N	N	N	N	N	N	N	N
	*bla*SHV-1	N	N	N	N	N	N	N	N	N	N	Y	N	N	N	N	N	N	N	N	N	N	N	N	N	N	N	N	N	N
	*bla*SHV-26	N	N	N	N	N	N	N	N	N	N	Y	N	N	N	N	N	N	N	N	N	N	N	N	N	N	N	N	N	N
	*bla*SHV-28	N	N	N	N	N	N	N	N	N	N	N	Y	Y	Y	Y	Y	Y	Y	Y	N	N	N	N	N	N	N	N	N	N
	*bla*SHV-36	N	N	N	N	N	N	N	N	N	N	N	N	N	N	N	N	N	N	N	N	N	Y	N	N	N	N	N	N	N
	*bla*SHV-40	N	N	N	N	N	N	N	N	N	Y	N	N	N	N	N	N	N	N	N	N	N	N	N	N	N	N	N	N	N
	*bla*SHV-56	N	N	N	N	N	N	N	N	N	Y	N	N	N	N	N	N	N	N	N	N	N	N	N	N	N	N	N	N	N
	*bla*SHV-78	N	N	N	N	N	N	N	N	N	N	Y	N	N	N	N	N	N	N	N	N	N	N	N	N	N	N	N	N	N
	*bla*SHV-79	N	N	N	N	N	N	N	N	N	Y	N	N	N	N	N	N	N	N	N	N	N	N	N	N	N	N	N	N	N
	*bla*SHV-85	N	N	N	N	N	N	N	N	N	Y	N	N	N	N	N	N	N	N	N	N	N	N	N	N	N	N	N	N	N
	*bla*SHV-89	N	N	N	N	N	N	N	N	N	Y	N	N	N	N	N	N	N	N	N	N	N	N	N	N	N	N	N	N	N
	*bla*SHV-94	N	N	N	N	N	N	N	N	N	N	N	N	N	N	N	N	N	N	N	Y	Y	N	N	N	N	N	N	N	N
	*bla*SHV-96	N	N	N	N	N	N	N	N	N	N	N	N	N	N	N	N	N	N	N	Y	Y	N	N	N	N	N	N	N	N
	*bla*SHV-98	N	N	N	N	N	N	N	N	N	N	Y	N	N	N	N	N	N	N	N	N	N	N	N	N	N	N	N	N	N
	*bla*SHV-106	N	N	N	N	N	N	N	N	N	N	N	Y	Y	Y	Y	Y	Y	Y	Y	N	N	N	N	N	N	N	N	N	N
	*bla*SHV-145	N	N	N	N	N	N	N	N	N	N	Y	N	N	N	N	N	N	N	N	N	N	N	N	N	N	N	N	N	N
	*bla*SHV-161	N	N	N	N	N	N	N	N	N	N	Y	N	N	N	N	N	N	N	N	N	N	N	N	N	N	N	N	N	N
	*bla*SHV-172	N	N	N	N	N	N	N	N	N	N	N	N	N	N	N	N	N	N	N	Y	Y	N	N	N	N	N	N	N	N
	*bla*SHV-179	N	N	N	N	N	N	N	N	N	N	Y	N	N	N	N	N	N	N	N	N	N	N	N	N	N	N	N	N	N
	*bla*SHV-187	N	N	N	N	N	N	N	N	Y	N	N	N	N	N	N	N	N	N	N	N	N	N	N	N	N	N	N	N	N
	*bla*SHV-194	N	N	N	N	N	N	N	N	N	N	Y	N	N	N	N	N	N	N	N	N	N	N	N	N	N	N	N	N	N
	*bla*SHV-199	N	N	N	N	N	N	N	N	N	N	Y	N	N	N	N	N	N	N	N	N	N	N	N	N	N	N	N	N	N
	*bla*TEM-1A	N	N	N	N	N	N	N	N	N	N	N	N	Y	N	N	N	N	N	N	N	N	N	N	N	N	N	N	N	N
	*bla*TEM-1B	N	N	Y	Y	Y	Y	Y	Y	Y	Y	Y	N	N	Y	Y	Y	Y	Y	Y	Y	Y	Y	N	N	N	N	N	N	N
	*bla*TEM-1C	N	N	N	N	N	N	N	N	N	N	N	N	N	N	N	N	N	N	N	N	N	Y	N	N	N	N	N	N	N
	*bla*VIM-2	N	N	N	N	N	N	N	N	N	N	N	N	N	N	N	N	N	N	N	N	N	N	Y	Y	Y	Y	Y	N	Y
	*fos*A	N	N	N	N	N	N	N	N	Y	Y	Y	Y	Y	Y	Y	Y	Y	Y	Y	Y	Y	N	Y	Y	Y	Y	Y	Y	Y
	*ere*A	N	N	N	N	N	N	N	N	N	N	Y	N	N	N	N	Y	N	N	N	N	N	N	N	N	N	N	N	N	N
	*mph*A	Y	Y	Y	Y	Y	Y	Y	Y	N	N	N	N	N	N	N	N	N	N	N	N	Y	Y	N	N	N	N	N	N	N
	*mdf*A	Y	Y	Y	Y	Y	Y	Y	Y	N	N	N	N	N	N	N	N	N	N	N	N	N	N	N	N	N	N	N	N	N
	*cat*A1	N	N	N	N	N	N	N	N	N	N	N	N	N	N	N	N	N	N	N	N	Y	N	N	N	N	N	N	N	N
	*cat*A2	N	N	N	N	N	N	N	N	Y	N	N	Y	Y	N	Y	Y	N	Y	N	Y	N	Y	N	N	N	N	N	N	N
	*cat*B3	Y	N	Y	Y	Y	Y	Y	Y	N	Y	Y	Y	Y	N	Y	Y	Y	N	Y	Y	Y	Y	N	N	N	N	N	N	N
	*cat*B7	N	N	N	N	N	N	N	N	N	N	N	N	N	N	N	N	N	N	N	N	N	N	Y	Y	Y	Y	Y	Y	Y
	*cml*A1	N	N	N	N	N	N	N	N	N	Y	Y	N	N	N	N	Y	N	N	N	N	N	N	N	N	Y	Y	Y	N	N
	*crp*P	N	N	N	N	N	N	N	N	N	N	N	N	N	N	N	N	N	N	N	N	N	N	Y	Y	Y	Y	Y	Y	Y
	*flo*R	N	N	N	N	N	N	N	N	N	N	N	N	N	N	N	N	N	N	Y	N	N	N	N	N	N	N	N	N	N
	*Oqx*A	N	N	N	N	N	N	N	N	Y	Y	Y	Y	Y	Y	Y	Y	Y	Y	Y	Y	Y	N	N	N	N	N	N	N	N
	*Oqx*B	N	N	N	N	N	N	N	N	Y	Y	Y	Y	Y	Y	Y	Y	Y	Y	Y	Y	Y	N	N	N	N	N	N	N	N
	*qnr*B1	N	N	N	N	N	N	N	N	N	Y	N	N	N	N	N	N	N	N	N	N	N	N	N	N	N	N	N	N	N
	*qnr*B6	N	N	N	N	N	N	N	N	N	N	N	N	N	Y	Y	Y	N	Y	N	N	N	N	N	N	N	N	N	N	N
	*qnr*S1	Y	N	N	Y	Y	Y	Y	Y	N	N	N	N	N	N	N	N	N	N	Y	N	N	N	N	N	N	N	N	N	N
	*ARR*-2	N	N	N	N	N	N	N	N	N	Y	Y	N	N	N	N	N	N	N	N	N	N	N	N	N	N	N	N	N	N
	*ARR*-3	N	N	N	N	N	N	N	N	N	N	N	N	N	Y	Y	N	N	Y	Y	N	N	N	N	N	N	N	N	N	N
	*ARR*-5	N	N	N	N	N	N	N	N	N	N	N	N	N	N	N	N	N	N	N	N	N	N	N	N	Y	N	Y	N	N
	*sul*1	N	Y	Y	Y	Y	Y	Y	Y	N	Y	Y	N	Y	Y	Y	Y	N	Y	Y	N	Y	Y	Y	Y	Y	Y	N	Y	Y
	*sul*2	Y	Y	Y	Y	Y	Y	Y	Y	Y	Y	Y	Y	Y	Y	Y	Y	Y	Y	Y	Y	Y	Y	N	N	N	N	N	N	N
	*tet*A	N	Y	Y	N	N	N	N	N	N	Y	N	N	N	N	N	N	N	N	N	Y	N	N	N	N	N	N	N	N	N
	*tet*B	N	N	N	Y	Y	Y	Y	Y	N	N	N	N	N	N	N	N	N	N	N	N	N	N	N	N	N	N	N	N	N
	*tet*D	N	N	N	N	N	N	N	N	N	N	Y	Y	Y	N	Y	Y	Y	Y	N	N	N	N	N	N	N	N	N	N	N
	*tet*G	N	N	N	N	N	N	N	N	N	N	N	N	N	N	N	N	N	N	N	N	N	N	N	N	Y	Y	Y	Y	N
	*dfr*A1	N	N	N	N	N	N	N	N	N	N	N	N	Y	N	N	N	N	N	N	N	N	N	N	N	N	N	N	N	N
	*dfr*A12	N	N	N	N	N	N	N	N	N	N	N	N	N	N	N	N	N	N	N	N	N	Y	N	N	N	N	N	N	N
	*dfr*A14	Y	N	N	N	Y	Y	Y	Y	N	Y	Y	Y	Y	N	N	N	Y	N	Y	Y	Y	N	N	N	N	N	N	N	N
	*dfr*A15	N	N	N	N	N	N	N	N	N	N	N	N	N	N	N	N	N	N	N	N	Y	N	N	N	N	N	N	N	N
	*dfr*A17	N	Y	Y	Y	Y	Y	Y	Y	N	N	N	N	N	N	N	N	N	N	N	N	N	N	N	N	N	N	N	N	N
	*dfr*A27	N	N	N	N	N	N	N	N	N	N	N	N	N	Y	Y	Y	N	Y	Y	N	N	N	N	N	N	N	N	N	N
	*dfr*A30	N	N	N	N	N	N	N	N	Y	N	N	N	N	N	N	N	N	N	N	Y	N	N	N	N	N	N	N	N	N
	*dfr*B5	N	N	N	N	N	N	N	N	N	N	N	N	N	N	N	N	N	N	N	N	N	N	N	N	Y	Y	Y	N	Y
	*Oqx*A	N	N	N	N	N	N	N	N	Y	Y	Y	Y	Y	Y	Y	Y	Y	Y	Y	Y	Y	N	N	N	N	N	N	N	N
	*Oqx*B	N	N	N	N	N	N	N	N	Y	Y	Y	Y	Y	Y	Y	Y	Y	Y	Y	Y	Y	N	N	N	N	N	N	N	N
	*qac*E	N	Y	Y	Y	Y	Y	Y	Y	N	Y	Y	N	Y	Y	Y	Y	N	Y	Y	N	Y	Y	Y	Y	Y	Y	Y	Y	Y
Plasmids	Col(BS512)	N	N	N	N	Y	Y	Y	Y	N	N	N	N	N	N	N	N	N	N	N	N	N	N	N	N	N	N	N	N	N
	Col(MG828)	Y	Y	Y	N	N	N	N	N	N	N	N	N	N	N	N	N	Y	N	Y	N	N	N	N	N	N	N	N	N	N
	Col156	N	Y	N	N	N	N	N	N	N	N	N	N	N	N	N	N	N	N	N	N	N	N	N	N	N	N	N	N	N
	Col440I	N	N	N	N	N	N	N	N	N	Y	Y	N	N	Y	N	Y	N	N	N	N	N	Y	N	N	N	N	N	N	N
	Col440II	N	N	N	N	N	N	N	N	N	N	N	Y	Y	N	N	N	N	N	N	N	N	N	N	N	N	N	N	N	N
	ColKP3	N	N	N	N	Y	Y	Y	Y	N	N	N	N	N	N	N	N	N	N	N	N	N	N	N	N	N	N	N	N	N
	ColRNAI	Y	Y	N	N	N	N	N	N	N	Y	N	N	Y	N	N	N	Y	N	Y	Y	N	N	N	N	N	N	N	N	N
	*Inc*A/C2	N	N	N	N	N	N	N	N	N	N	N	N	N	N	N	N	N	N	Y	N	N	N	N	N	N	N	N	N	N
	*Inc*B/O/K/Z	N	N	N	N	Y	Y	Y	Y	N	N	N	N	N	N	N	N	N	N	N	N	N	N	N	N	N	N	N	N	N
	*Inc*FIA	Y	Y	Y	Y	Y	Y	Y	Y	N	N	N	N	N	N	N	N	N	N	N	N	N	N	N	N	N	N	N	N	N
	*Inc*FIA (HI1)	N	N	N	N	N	N	N	N	Y	N	Y	N	N	N	Y	Y	N	Y	N	N	N	N	N	N	N	N	N	N	N
	*Inc*FIB (AP001918)	Y	Y	Y	Y	Y	Y	Y	Y	N	N	N	N	N	N	N	N	N	N	N	N	N	N	N	N	N	N	N	N	N
	*Inc*FIB (K)	N	N	N	N	N	N	N	N	N	Y	Y	Y	Y	Y	Y	Y	Y	Y	Y	Y	Y	N	N	N	N	N	N	N	N
	*Inc*FIB (Mar)	N	N	N	N	N	N	N	N	N	N	N	N	N	N	N	N	N	N	N	N	Y	Y	N	N	N	N	N	N	N
	*Inc*FIB (pQil)	N	N	N	N	N	N	N	N	N	N	N	N	N	N	Y	N	N	N	N	N	Y	N	N	N	N	N	N	N	N
	*Inc*FII	Y	N	Y	N	N	N	N	N	N	Y	N	N	N	N	N	N	N	N	N	Y	N	N	N	N	N	N	N	N	N
	*Inc*FII (K)	N	N	N	N	N	N	N	N	Y	Y	Y	Y	Y	Y	Y	Y	Y	Y	Y	Y	Y	N	N	N	N	N	N	N	N
	*Inc*FII (pAMA1167-NDM-5)	N	N	N	Y	Y	Y	Y	Y	N	N	N	N	N	N	N	N	N	N	N	N	N	N	N	N	N	N	N	N	N
	*Inc*FII (pCoo)	N	N	N	N	Y	Y	Y	Y	N	N	N	N	N	N	N	N	N	N	N	N	N	N	N	N	N	N	N	N	N
	*Inc*FII (pCRY)	N	N	N	N	N	N	N	N	N	N	N	Y	N	N	N	N	N	N	N	N	N	N	N	N	N	N	N	N	N
	*Inc*FII (pKPX1)	N	N	N	N	N	N	N	N	N	N	N	N	N	N	N	N	N	N	N	N	N	Y	N	N	N	N	N	N	N
	*Inc*FII (pRSB107)	N	Y	N	N	Y	Y	Y	Y	N	N	N	N	N	N	N	N	N	N	N	N	N	N	N	N	N	N	N	N	N
	*Inc*HI1B	N	N	N	N	N	N	N	N	N	N	N	N	N	N	N	N	N	N	N	N	Y	N	N	N	N	N	N	N	N
	*Inc*l1	Y	N	N	N	N	N	N	N	N	N	N	N	N	N	N	N	N	N	N	N	N	N	N	N	N	N	N	N	N
	*Inc*N	N	N	N	N	N	N	N	N	N	N	N	N	N	N	N	N	N	N	Y	N	N	N	N	N	N	N	N	N	N
	*Inc*Q1	N	N	N	Y	N	N	N	N	N	N	N	N	N	N	N	N	N	N	N	Y	N	N	N	N	N	N	N	N	N
	*Inc*R	N	N	N	N	N	N	N	N	Y	N	Y	Y	Y	Y	Y	Y	N	Y	N	Y	Y	N	N	N	N	N	N	N	N
	*Inc*X3	N	N	N	N	Y	Y	Y	Y	N	N	N	N	N	N	N	N	N	N	N	N	N	N	N	N	N	N	N	N	N
	*Inc*X4	Y	N	N	N	Y	Y	Y	Y	N	N	N	N	N	N	N	N	N	N	N	N	N	N	N	N	N	N	N	N	N
	p0111	N	N	Y	N	N	N	N	N	N	N	N	N	N	N	N	N	N	N	N	N	N	N	N	N	N	N	N	N	N

Green colour/N = No; Red colour/Y = Yes; GA = Gauteng; KZ = KwaZulu Natal; WC = Western Cape; A = Adult ward; P = Paediatric ward; U = Unknown ward.

**Table A2:** Antibiotic susceptibility profiles of 8 *Escherichia coli*, 14 *Klebsiella pneumoniae*, and 7 *Pseudomonas aeruginosa* blood culture isolates selected for WGS.

		Amoxicillin/Clavulanate Acid n = (%)	Ceftazidime/Avibactam n = (%)	Ceftolozane/Tazobactam n = (%)	Piperacillin/Tazobactam n = (%)	Cefotaxime n = (%)	Ceftazidime n = (%)	Aztreonam n = (%)	Ertapenem n = (%)	Imipenem n = (%)	Meropenem n = (%)	Colistin n = (%)	Gentamicin n = (%)	Tobramycin n = (%)	Amikacin n = (%)	Ciprofloxacin n = (%)	Trimethoprim/Sulfamethoxazole n = (%)
*E. coli* (n = 8)	S	1 (13)	6 (75)	0 (0)	1 (13)	0 (0)	0 (0)	0 (0)	0 (0)	3 (38)	3 (38)	0 (0)	4 (50)	0 (0)	3 (38)	0 (0)	0 (0)
	I	0 (0)	0 (0)	0 (0)	0 (0)	0 (0)	0 (0)	0 (0)	0 (0)	1 (13)	0 (0)	7 (77)	0 (0)	0 (0)	2 (25)	0 (0)	0 (0)
	R	7 (88)	2 (25)	8 (100)	7 (88)	8 (100)	8 (100)	8 (100)	8 (100)	4 (50)	5 (63)	1 (13)	4 (50)	8 (100)	3 (38)	8 (100)	8 (100)
*K. pneumoniae* (n = 14)	S	0 (0)	14 (100)	0 (0)	1 (7)	0 (0)	0 (0)	0 (0)	14 (100)	14 (100)	14 (100)	0 (0)	0 (0)	1 (7)	14 (100)	2 (14)	0 (0)
	I	0 (0)	0 (0)	0 (0)	1 (7)	0 (0)	0 (0)	0 (0)	0 (0)	0 (0)	0 (0)	13 (93)	0 (0)	2 (14)	0 (0)	1 (7)	0 (0)
	R	14 (100)	0 (0)	14 (100)	12 (86)	14 (100)	14 (100)	14 (100)	0 (0)	0 (0)	0 (0)	1 (7)	14 (100)	11 (79)	0 (0)	11 (79)	14 (100)
*P. aeruginosa* (n = 7)	S	0 * (0)	0 (0)	0 (0)	0 (0)	0 * (0)	0 (0)	6 (84)	0 * (0)	0 (0)	0 (0)	0 (0)	1 (14)	0 (0)	0 (0)	0 (0)	0 * (0)
	I	0 * (0)	0 (0)	0 (0)	1 (14)	0 * (0)	0 (0)	1 (14)	0 * (0)	0 (0)	0 (0)	4 (57)	1 (14)	0 (0)	0 (0)	0 (0)	0 * (0)
	R	0 * (0)	7 (100)	7 (100)	6 (86)	0 * (0)	7 (100)	0 (0)	0 * (0)	7 (100)	7 (100)	3 (43)	5 (71)	7 (100)	7 (100)	7 (100)	0 * (0)

S = Susceptible; I = Intermediate; R = Resistant; * = Antibiotic MIC breakpoints not covered in the 2021 Clinical Laboratory Standards Institute (CLSI) guidelines.
